# Chemical modification of selenium-containing amino acids caused by non-thermal dielectric-barrier discharge atmospheric-pressure plasma†

**DOI:** 10.1039/d4ra05754f

**Published:** 2024-11-29

**Authors:** Fahd Afzal, Dariusz Śmiłowicz, Friederike Kogelheide, Anna Lena Schöne, Katharina Stapelmann, Peter Awakowicz, Nils Metzler-Nolte

**Affiliations:** a Inorganic Chemistry I, Bioinorganic Chemistry, Ruhr University Bochum 44780 Bochum Germany nils.metzler-nolte@rub.de; b Department of Chemistry, University of Wisconsin–Madison Madison Wisconsin 53706 USA; c Institute for Electrical Engineering and Plasma Technology, Ruhr University Bochum 44780 Bochum Germany; d Department of Nuclear Engineering, North Carolina State University Raleigh North Carolina 27695 USA

## Abstract

Since non-thermal atmospheric-pressure (“cold”) plasma sources, such as the dielectric-barrier discharge (DBD), have appeared to be remarkably active in wound healing medicine, the elucidation of cold plasma safety and possible secondary undesirable effects becomes of paramount importance. Selenium-containing amino acids, which are commonly incorporated in many enzymes, came in the spotlight for elucidating the plasma impact as easily oxidizable natural targets. The scope of this study was to analyse the impact of non-thermal plasma on selenium-containing amino acids. Moreover, this research examines the emerging role of metals in the context of oxidation potency of reactive species generated by plasma. The dielectric barrier discharge (DBD) was used to treat 4 mM solutions of Se-(methyl)seleno-l-cysteine (1), l-selenomethionine (2) and seleno-l-cystine (3) for varying treatment times to investigate possible degradation products. In this study we used two redox active iron complexes as well as a redox inert zinc complex in order to compare their capacity to affect chemical modifications caused by plasma. The solutions with selenium-containing amino acids after plasma treatment were analyzed by IR spectroscopy, electrospray ionization mass spectrometry (ESI-MS) and High Performance Liquid Chromatography (HPLC). Several oxidation products were observed as a consequence of plasma treatment, namely: Se-(methyl)seleno-l-cysteine (1) and l-selenomethionine (2) were oxidized to selenoxide and selendioxide derivatives, wheres the Se–Se dimer, seleno-l-cystine (3), was converted to Se-cysteine and seleninic acid. Additionally and to our surprise, redox active iron(ii) and iron(iii) complexes as well as the non-redox active zinc(ii) complex caused the same oxidation pattern when added to the plasma treatment mixtures. Finally, a comparison of the results from Se-containing amino acids with those of their S-containing counterparts revealed that Se-containing amino acids are less prone to cold plasma oxidation than S-containing molecules. By elucidating molecular details of plasma–biomolecule interactions herein we aim to contribute to a better understanding of the complex beneficial medical effects of cold plasma treatments.

## Abstract 2

Herein, we present an investigation of the chemical modifications of selenium-containing amino acids which are caused by non-thermal plasma in the presence of redox active iron complexes as well as a redox inert zinc complex.

## Graphical abstract

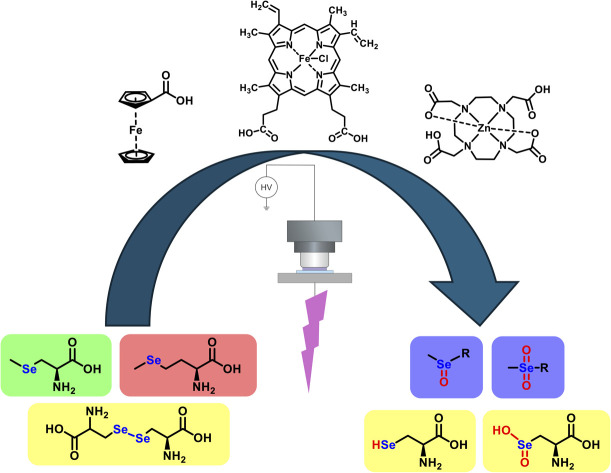

## Introduction

Since recognition of selenium as an essential trace element for animals and humans in 1957, extensive investigations into the physiological chemistry of selenium have began.^1,2^ Selenium in mammalians is mainly found in two organoselenium compounds: selenocysteine (Sec, U) and selenomethionine (SeMet), structurally identical to cysteine and methionine, but possessing selenium instead of sulphur.^3,4^ Selenium-containing amino acids appear to be better nucleophiles, electrophiles and leaving groups than their sulphur-containing equivalents.^5–7^ These features lead to higher chemical reactivity in comparison to parent amino acids.^8,9^ Consequently, there is no free selenocysteine or selenomethionine inside cells in order to avoid uncontrolled NADPH oxidation and reactive oxygen species formation.^10–12^ Both selenium-containing amino acids are incorporated into antioxidant, cellular protective enzymes, such as thioredoxin reductase (TrxR), glutathione peroxidase (GPx) and iodothyrosine deiodinases (DIO) with selenocysteine in an active-site, C-terminal redox center –Gly–Cys–Sec–Gly–COOH, crucial for catalytic activity.^13–15^ The presence of such selenoenzymes, commonly encountered in mammalian cells, give the advantage in the form of broader range of substrates and conditions in which enzyme activity is possible, over ones containing sulphur in bacterial cells.^16–18^

Since non-thermal plasma has become a successful tool in redox-based healing therapies, *e.g.* wound healing, cancer, and inflammatory diseases,^19,20^ eukaryotic cells and all their components became natural targets for elucidating the effects induced by plasma treatment.^21,22^ Reactive oxygen species (ROS) and reactive nitrogen species (RNS) created during plasma treatment are regarded as the *raison d'etre* responsible for antimicrobial activity of cold plasma. On the other hand, they cause irreversible chemical modifications of biomolecules.^23,24^ Literature provides us with information about the decomposition products of several biomolecules when exposed to non-thermal atmospheric pressure plasma.^25^ Studies involving amino acids showed that cold plasma causes preferentially hydroxylation and nitration of aromatic rings, sulfonation and disulfide linkage formation of thiol groups in cysteine and sulfoxidation of methionine.^26^ Plasma treatment of the disaccharide sucrose resulted in the cleavage of the glycosidic bond after 5 min,^27^ whereas monosaccharides, such as ribose and glucose, were decomposed to formic acid, glycolic acid, glyceric acid, tartronic acid, tartaric acid, acetic acid, and oxalic acid after direct exposure to DBD plasma.^28^ Non-thermal plasma was also successfully used to decontaminate wastewater containing toxic pesticides or antibiotics.^29^ The degradation of hydrogen cyanide in distilled water as well as in industrial wastewaters to NO_3_^−^, NO_2_^−^, NH_4_^+^, N_2_, HCO_3_^−^, CO_3_^−^ was observed, however within a long exposure time of 90 min.^30^ Cold plasma caused the oxidation of atrazine in contaminated water to simazine amide, atrazine amide, deethylatrazine and to the deeper oxidation product didealkylatrazine within 15 min.^31^ Plasma generated by a DBD appeared to be able to degrade methyl orange (MO) in organic dye wastewater, by breaking down the diazo group (–N=N–) within 10 min.^32^ Also removal of ibuprofen from aqueous solutions by oxidation to aliphatic carboxylic acids within 15 min was observed.^33^

Transition metals play indispensable roles in the human body from being a vital component in many enzymes, taking part in bone, tissues and red blood cells formation, to neurosignalling and DNA regulation.^34,35^ Among the wide range of essential transition metals two groups can be distinguished: redox active and non-redox active (or redox inactive) metals.^36^ Iron belongs to the first group and is highly abundant in the human body. It occurs mainly in two oxidation states, *i.e.* in reduced ferrous form (Fe^2+^) and oxidized ferric (Fe^3+^) form.^37^ The average adult human body contains approximately 4.5 g of Fe. In addition to its role in oxygen transport, iron is also vital in dioxygen activation reactions and takes part in the production of ROS through the Fenton redox reaction.^38^ The second group with non-redox active elements is perhaps best represented by zinc, with an abundance of 3.2 g in the human body.^39,40^ Zinc exists solely as the positively charged ion Zn^2+^ and is a binding component of many enzymes, such as transcription factors or albumin.^41,42^

Despite the success of cold plasma therapy in wound healing, much uncertainty still exists about the relation between activity and interactions of cold plasma with biological molecules.^43^ It is essential to investigate and explain the effects of cold plasma on cell components at the molecular level.^44^ Especially, very little is currently known about the impact of plasma on biomolecules in the environment mimicking the presence of cell components, particularly containing biologically relevant metals.^45^ In our previous research, we have focussed on the influence of cold plasma on biologically relevant molecules, such as glutathione (GSH), cysteine and cyanocobalamin.^46,47^ While the exact mechanisms underlying the interaction between plasma and compounds in solution remains largely unknown, it is hypothesized that plasma-generated reactive oxygen species (ROS) are responsible for the oxidative changes observed in the compound. For example, cold plasma caused chemical modification of GSH and GSSG resulting in many oxidation species. Also, the decomposition of cyanocobalamin with concomitant loss of catalytic activity within treatment times longer than 2 min was observed. Moreover, our previous research recognises the critical role played by iron complexes on the distribution of oxidation products caused by cold plasma.^48^ Our results demonstrated that using redox active metal complexes influences the nature and the relative amounts of oxidation products.^49^

**Fig. 1:**
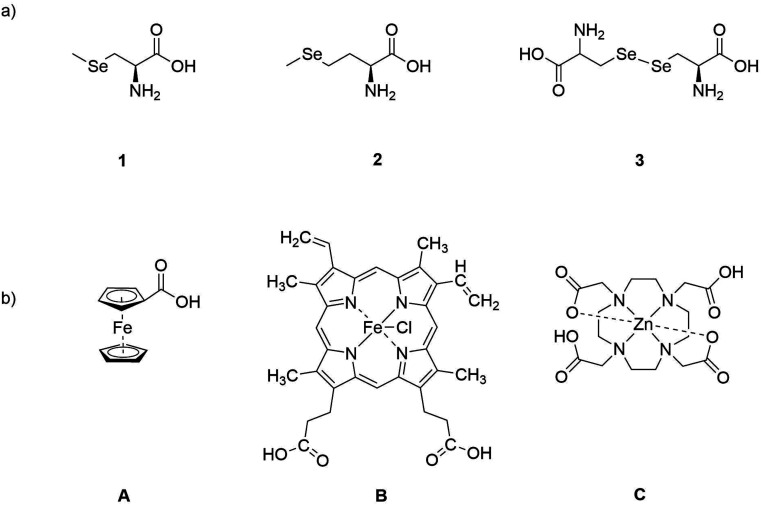
Structures of (a) selenium-containing substrates 1–3, (b) metal complexes: iron(ii) ferrocenecarboxylic acid (A), iron(iii) hemin (B) and zinc(ii) DOTA complex (C).

There are two primary aims of this study: (i) to investigate the influence of DBD plasma on selenium-containing amino acids, with focus on the plasma generated ROS, as the main effect of cold plasma, and (ii) to ascertain the impact of metal complexes containing redox active and redox inactive metals in chemical modifications caused by cold plasma. Se-containing compounds 1, 2 and 3 will be examined as model compounds (Fig. 1a). Since organic selenium compounds are among the most easily oxidized simple biomolecules, they appear to be obvious molecular targets for the ROS generated by cold plasma. As redox active complexes, we utilize the iron(ii) complex ferrocenecarboxylic acid (complex A), the chloro(protoporphyrinato)iron(iii) (hemin) (complex B), and finally the ZnDOTA (complex C) as redox-inactive zinc(ii) complex (Fig. 1b). All experiments were performed within an analytical setting and with very small quantities. Purification and the subsequent application of analytical methods such as ^1^H-NMR or even structural characterization by X-ray crystallography are challenging, as these techniques often require a larger amount of sample material to yield meaningful data. Therefore, this study focuses on IR spectroscopy, electrospray ionization mass spectrometry (ESI-MS) and High Performance Liquid Chromatography (HPLC) as analytical methods.

## Results

### Stability of substrates and zinc(ii) complex

First of all, the stability of selenium-containing substrates in distilled water was monitored by electrospray ionization mass spectrometry (ESI-MS). Compound 1, when ionized in negative mode, gave an ESI-MS signal corresponding to [M − H]^−^ at *m*/*z* = 182. Compound 2 gave a signal in negative mode corresponding to [M − H]^−^ at *m*/*z* = 196. The mass spectrum of compound 3 displayed one signal on the positive mode corresponding to the sodium adduct [M + Na]^+^ at *m*/*z* = 357. All selenium-containing amino acids showed the same, stable ionization pattern after 1, 3, 5 and 20 min. The *m*/*z* values are consistent with the expected isotopic mass distribution pattern of selenium. The mass spectrum of zinc(ii) complex C presents one signal with moderate intensity at *m*/*z* = 466 corresponding to the [M + H]^+^ moiety and another signal with high intensity corresponding to the [M + Na]^+^ at *m*/*z* = 489 (see ESI Fig. S4 and S5†). To monitor the stability of substrates and metal complexes also HPLC analysis was involved. For each selenium-containing molecule the HPLC trace showed only one peak at 6.6 min, 7.3 min and 11.5 min, respectively (see ESI Fig. S11–S13†). The HPLC chromatograms of the zinc(ii) complex revealed one signal at 9.1 min. Neither HPLC profiles nor ESI-MS spectra for all substrates were affected within the extended incubation time. Also the Zn(ii)DOTA complex appeared to be stable within 24 h. The stability of the iron complexes in aqueous solution was confirmed by us previously.^49^ Both iron complexes A and B, zinc(ii) complex C, and all selenium-containing amino acids exhibited high stability in aqueous medium with no traces of decomposition, which makes them suitable candidates for our studies described in the following.

### Interactions of metal complexes with substrates

To monitor whether the metal complexes may cause any modification of the seleno amino acids by themselves, the interactions between these complexes and the substrates were investigated. Corresponding substrate and metal complexes were mixed in ratio 1 : 1 and dissolved in distilled water. Samples containing substrates and metal complexes were incubated for 1, 3, 5 and 20 min and subsequently analyzed by HPLC and ESI-MS. The HPLC chromatogram for the compound 1 showed two signals, one at 5.1 min and the second depending on the metal complex used for incubation, namely at 7.1 min (A), at 7.5 min (B) and at 9.1 min (C). The same HPLC profile was obtained for substrates 2 and 3 with corresponding signals for selenium compounds at 5.6 min and at 6.8 min. The HPLC traces showed the same, stable patterns after 1, 3, 5 and 20 min. The negative-ion ESI mass spectra of compound 1 after 1, 3, 5 and 20 min of incubation with complex A showed two signals at *m*/*z* = 182 and at *m*/*z* = 229, corresponding to the molecular masses of compound 1 and the ferrocenecarboxylic acid, respectively. The mass spectra after incubation with complex B and with complex C revealed the presence of signals at *m*/*z* = 611 and at *m*/*z* = 467, corresponding to the molecular masses of the hemin complex and the zinc(ii) DOTA complex, respectively. The mass spectrum measured in the negative ionization mode for compounds 2 and 3 showed signals at *m*/*z* = 196 and at *m*/*z* = 334 corresponding to the [M − L]^−^ species of unaffected compounds 2 and 3, respectively, in addition to the signals from the metal complexes. The incubation experiments hence underscored that none of the metal complexes had any influence on selenium-containing amino acids. The full-scan mass spectra and HPLC traces are available in the ESI.†

### Influence of plasma on zinc complex

Secondly, the time-dependent impact of DBD treatment on the ZnDOTA complex C was investigated. The mass spectrum of complex C treated with plasma for 1 min and 3 min displayed the same ionization pattern with signals at *m*/*z* = 466 and at *m*/*z* = 489 corresponding to the [M + H]^+^ and [M + Na]^+^, as the spectrum for the untreated complex (see ESI Fig. S4 and S5†). The HPLC analysis confirms the full stability of complex C (see Fig. S3 and S6†). However, some changes in the fragmentation pattern were observed after 5 min of plasma treatment. Besides the two characteristic signals, a new one was observed at *m*/*z* = 432 with moderate intensity corresponding to [M − 2OH_2_]^+^. This signal was assigned to complex C after loosing two H_2_O molecules. Extending the treatment time to 20 min caused no further changes in the fragmentation pathway. The impact of plasma on iron complexes was investigated by us previously and reported.^49^ In conclusion, both iron complexes and the zinc complex exhibit high stability in aqueous medium during plasma treatment, which makes them suitable candidates for the cold plasma studies.

### Influence of cold plasma on selenium-containing amino acids

**Fig. 2:**
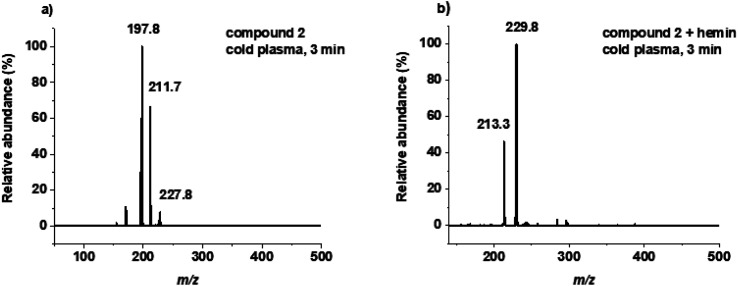
ESI-MS spectra of (a) compound 2 after plasma treatment alone, and (b) compound 2 after plasma treatment in the presence of iron complex.

The negative-ion ESI-MS of substrate 1, the most common physiological selenide and close analogue to cysteine, showed several signals after 1 min of plasma treatment. The first signal at *m*/*z* = 182, which corresponds to the [M − H]^−^ species, is identified as unconverted substrate. The signal at *m*/*z* = 197 was assigned to an oxidized form of compound 1 carrying a selenoxide moiety. The third signal at *m*/*z* = 211 was identified as selenocysteine possessing a selendioxide moiety. ESI-MS spectra after 3, 5 and 20 min showed the presence of the same species. The negative ionization mode mass spectrum for compound 2 after 3 min of plasma treatment also showed a variety of products. Among them, signals at *m*/*z* = 198, *m*/*z* = 212 and at *m*/*z* = 228 were identified as unmodified substrate, selenomethionine with selenoxide moiety and selenomethionine with selendioxide moiety (Fig. 2a). Extending the plasma treatment time increased the number of oxidation products according to ESI-MS measurements. HPLC analysis of selenium-containing molecules 1 and 2 after plasma treatment confirmed the results from mass spectrometry (see ESI Fig. S14†). Treatment of compound 1 with the DBD revealed a mixture of products existing in the solution. Signals at 5.1 min, at 5.3 min and at 5.7 min were assigned to compound 1, species with selenoxide moiety and species with selendioxide moiety, respectively. A similar HPLC profile was obtained for compound 2 for which, among the mixture of products, signals at 5.6 min, at 5.9 min and at 6.1 min were identified. The behaviour of compound 1 and 2 during DBD cold plasma treatment is reminiscent of that shown for *N*-acetylcysteine (NAC), resulting in the wide range of oxidized products.^50^

**Fig. 3:**
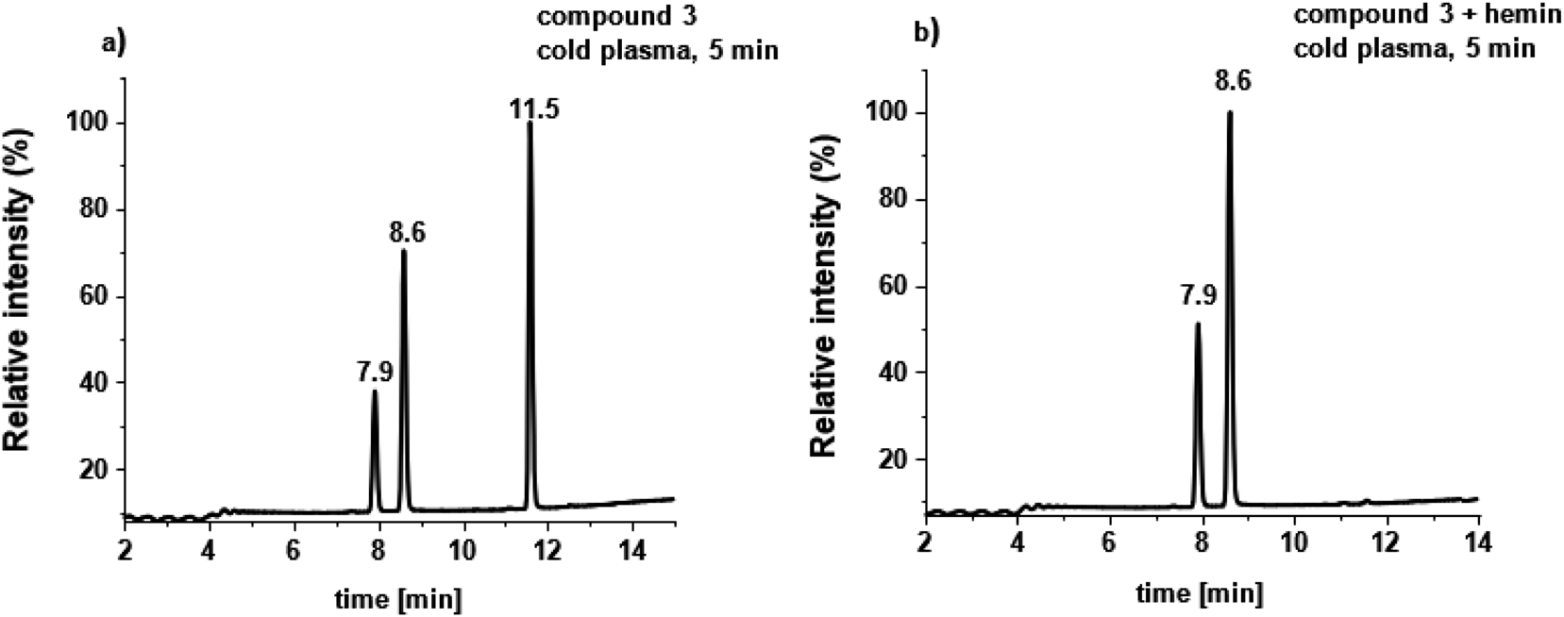
HPLC traces of (a) compound 3 after plasma treatment alone, and (b) compound 3 after plasma treatment in the presence of iron complex.

Finally, substrate 3, the diselenide analogue (R_2_Se_2_) of disulfides^51^ was treated with cold plasma. Already after 1 min of plasma treatment the mass spectrum revealed significant changes in comparison to the untreated samples. The signal at *m*/*z* = 168 corresponding to a [M + H]^+^ adduct was identified as Se-cysteine, (RSeH, analogue of thiols), the cleavage product of Se–Se bond. Also, seleninic acid was observed as an oxidation product at *m*/*z* = 223 as [M + Na]^+^ species (see ESI Fig. S15†). The samples after 3, 5 and 20 min of treatment showed the presence of the same species. HPLC traces after plasma treatment of compound 3 showed three peaks, one at 7.9 min assigned to Se-cysteine, and a second signal with retention time at 8.6 min corresponding to seleninic acid (Fig. 3a). Also the signal confirming the presence of the unconverted substrate was observed (11.5 min). Our findings correlate with data found in literature, where *in situ* generated reactive oxygen species cleaved diselenide bonds within 3,3′-diselanediyldipropionic acid, in the core-cross-linked (CCL) micelles.^52^

**Fig. 4:**
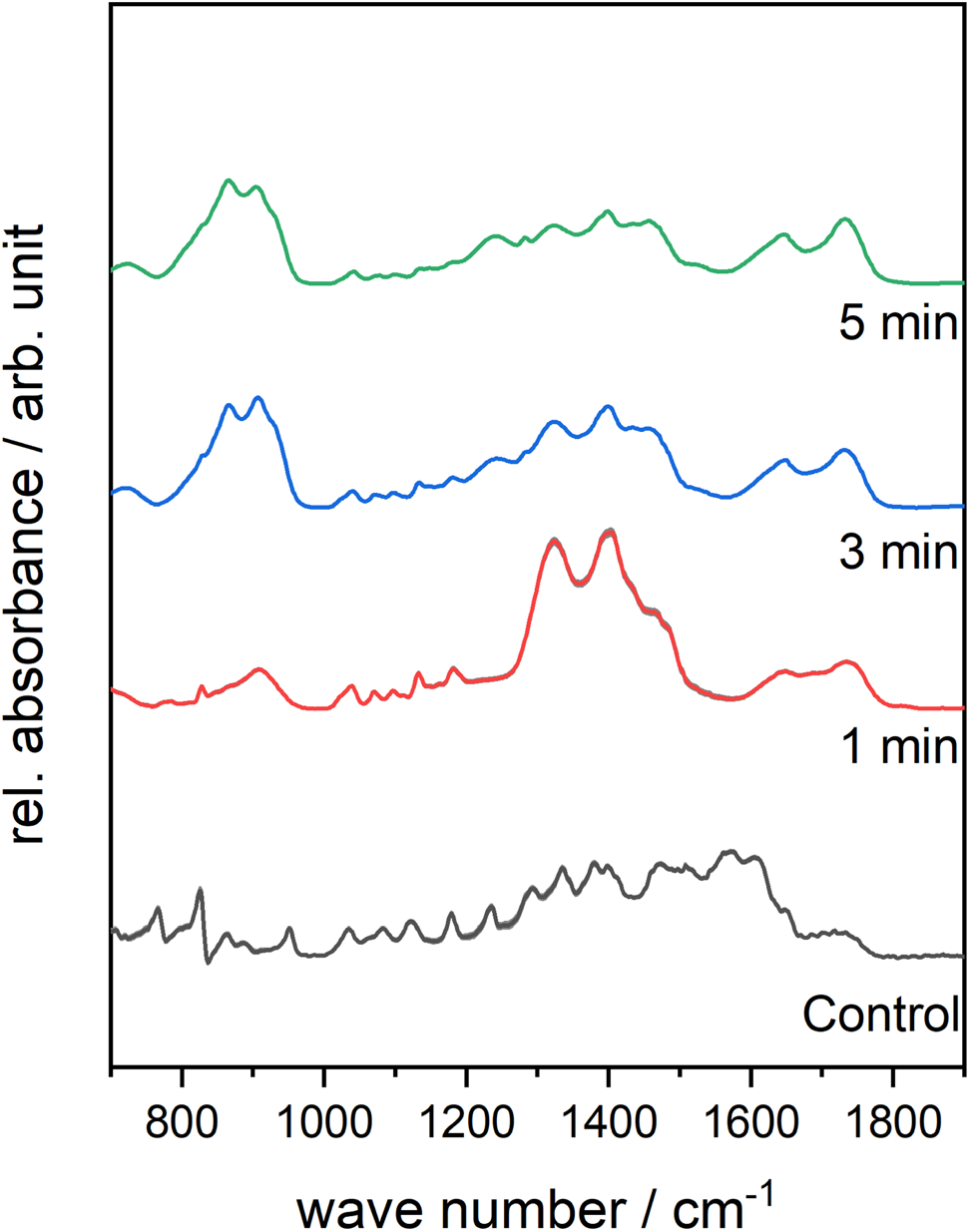
Mean FTIR-spectra of plasma-treated compound 3 within the range of 700–1900 cm^−1^ as a function of different treatment times.

IR analysis was further used to confirm the breakdown of selenium–selenium bonds and possible further oxidation of selenium moieties. Fig. 4 presents the FTIR-spectra of plasma-treated compound 3 within the range of 700–1900 cm^−1^ in order to follow the crucial Se–O changes after oxidation of Se. The full IR spectra are available in the ESI (Fig. S23–S25†). In the FTIR spectra of compound 3 after 1 min of plasma treatment, a new broad band in the 920–820 cm^−1^ range appeared. After 3 min, two distinctive bands in this range appeared at 880 cm^−1^ and at 850 cm^−1^. These bands are assigned to the asymmetric and the symmetric stretching vibrations of the Se=O bonds with double-bond character, respectively. Extending the treatment time to 5 min caused no further major changes in oxidation pattern according to the IR studies.

### Influence of cold plasma on selenium-containing amino acids in the presence of iron complexes

The behaviour of 1 after plasma treatment in the presence of the iron complex A in ESI-MS analysis is as follows: signals at *m*/*z* = 197 and at *m*/*z* = 212 corresponding to [M − H]^−^ ions were observed. The first signal is assigned to the oxidized form of compound 1 with selenoxide moiety, the second to the further oxidized product with selendioxide moiety. No molecular signal of compound 1 was found. Extending the incubation time to 20 min caused no further modification. Samples containing complex B exhibited the same oxidation products. The negative-ion ESI mass spectrum of compound 2 in the presence of the iron(ii) complex A shows the formation of [M − H]^−^ species at *m*/*z* = 212 and at *m*/*z* = 228 (Fig. 2b). Both are assigned to the stepwise oxidation products of compound 2. Extending the treatment time to 3, 5 and 20 min confirmed the presence of the same oxidation products. Negative-ion ESI mass spectrum of compound 3 in the presence of iron complexes after plasma treatment gave a signal at *m*/*z* = 200 as the dominant ion, corresponding to the seleninic acid. The ESI-MS spectrum also showed a low intensity signal at *m*/*z* = 168 which is ascribed to Se-cysteine. The same species were detected after 3, 5 and 20 min of plasma treatment. The parent compound 1, 2 and 3 were not observed anymore in the samples after DBD plasma treatment in the presence of iron(ii) complex A as well as iron(iii) complex B. HPLC traces of samples after plasma treatment in the presence of either iron complex differ from those without iron complexes present (Fig. 3b). The HPLC chromatogram for compound 3 showed two signals, one at 7.9 min assigned to the Se-cysteine molecule, second at 8.6 min assigned to seleninic acid. The HPLC traces for compounds 2 and 3 confirmed the presence of two oxidation products in the solution. The results of plasma-treated compounds 3, reflect those of Smith *et al.*, where cysteic acid was observed as one of the cysteine oxidation products.^27^

### Influence of cold plasma on selenium- and sulfur-containing amino acids in the presence of zinc complex

Additionally, compound 3 was treated with cold plasma in the presence of a non-redox active metal complex, namely the zinc(ii) complex C. The ESI-MS spectrum after 1 min of plasma treatment shows ions at *m*/*z* = 168 and at *m*/*z* = 334 corresponding to [M − H]^−^ species of Se-cysteine and unaffected substrate, respectively. After 3 min, additionally to those two products, a signal at *m*/*z* = 200 was observed and assigned to seleninic acid. The ESI-MS spectrum for samples with plasma treatment extended to 5 min showed the same distribution of products. After 20 min of plasma treatment, only the molecular ions at *m*/*z* = 168 and at *m*/*z* = 200 corresponding to [M − H]^−^ species of Se-cysteine and seleninic acid were present.

Since this zinc complex was used for first time in our plasma research, we also investigated the influence of cold plasma on the glutathione dimer GSSG in the presence of zinc(ii) complex C. GSSG was treated with DBD for 1, 3 and 5 min in the presence of Zn(ii)DOTA. After 1 min of plasma treatment, the HPLC profile exhibited the presence of only one species and follow-up ESI-MS analysis confirmed the presence of species at *m*/*z* = 354 assigned to [M − H]^−^ glutathione sulfonic acid GSO_3_H (data available in the ESI Fig. S17–S22†). Already after 1 min of plasma treatment, all GSSG molecules were converted to glutathione sulfonic acid GSO_3_H. Extending the treatment time showed the same oxidation pattern. These results clearly show that zinc(ii) complex C exerts the same influence on oxidation products as the iron(ii) and iron(iii) complexes. In comparison to our previous studies,^49^ there is no difference between using iron complex or zinc complex in product distribution. Taken together, the above experiments complete the picture of influence of cold plasma on S–S and Se–Se containing small molecules in the presence of redox active and non-redox active metal complexes.

## Discussion

**Fig. 5:**
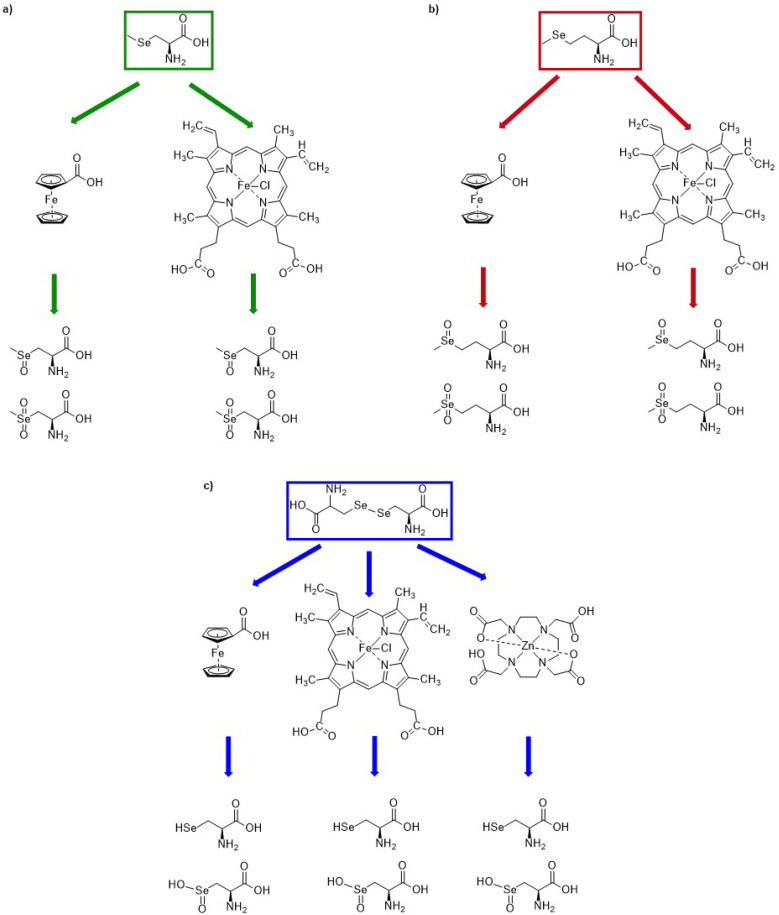
Chemical modifications of (a) compound 1, (b) compound 2 and (c) compound 3 after plasma treatment in the presence of metal complexes.

The main goal of the current study was to determine the impact of DBD plasma on selenium-containing amino acids. IR spectroscopy, ESI mass spectrometry and High Performance Liquid Chromatography (HPLC) were the methods used to identify the modifications induced by cold plasma. Our data show that Se-containing compounds 1, 2 and 3 were oxidized to a range of products. Among them, oxidation compounds with selenoxide and selendioxide moieties derived from compound 1 and 2 were detected as the main species. After plasma treatment of seleno-l-cystine, the Se-cysteine molecule and seleninic acid along with unconverted starting material were present in the solution. The distribution of the products in solution suggests a plasma-driven time-dependent oxidation of selenium-containing amino acids. During our prior studies, we have reported the importance of iron complexes in plasma-caused chemical modifications of GSH and GSSG. DBD plasma in the presence of iron(ii) and iron(iii) complexes converted them selectively into GSO_3_H, whereas without metal complexes to the variety of S-containing products.^46,49^ Building on those previous findings, the second aim of this study was to investigate the effects of the cold plasma on selenium-containing amino acids in the presence of redox active iron complexes. Characterisation of metal contributions is important for our advanced understanding of the role of cold plasma on biological systems, given that metals like Fe and Zn are ubiquitous in almost any biological system. In the presence of iron(ii) ferrocenecarboxylic acid (A) and iron(iii) hemin complex (B) the distribution of products differs from those obtained from plasma-treated selenium-containing amino acids alone. In comparison to the mixture of oxidation products, the presence of iron complexes during plasma treatment resulted in a reduced amount of oxidation products, giving only selenoxide and selendioxide moieties derived from compound 1 and 2, and only Se-cysteine and seleninic acid derived from compound 3 as the only product after 5 min of plasma treatment (Fig. 5). In the final part of this study, instead of redox active iron complexes, the non-redox zinc(ii) complex was used. Using complex C essentially gave the same results, however typically after longer treatment time. This can be rationalized by the fact that the metal complexes mainly serve as Lewis acids, a (possible) redox cycling of the iron complexes on the other hand does not seem to play a major role. Our results lead to a deeper understanding of the effects of plasma treatment on biomolecules in solution. In general, non-thermal plasma treatment clearly causes chemical modifications of selenium-containing amino acids together with a plasma-related cleavage of the selenium–selenium dimer of cystine. Our data together with the recently published studies about cold plasma effects on proteins, DNA, lipids and carbohydrates clearly confirm the plasma-induced time-dependent chemical alterations to the biologically active molecules.^44,53–55^ However, the mechanism of action between plasma and compound remains still unknown. Further studies will be carried out in order to elucidate the impact of cold plasma generated by the DBD on the catalytic activity of selenoenzymes.

## Materials and methods

### Experimental setup

The experimental setup used in this work has been described in a similar way before.^46,49^ The experiments in this study were carried out with a dielectric barrier discharge, which consists of a copper electrode covered with aluminium oxide (Al_2_O_3_) with a thickness of 1 mm. The electrode has a diameter of 10 mm and the distance between the driven electrode and the sample was kept constant at 1 mm. The samples were placed on a grounded aluminium plate and ambient air was used as the process gas. The temperature in the lab was adjusted to 20 °C and the relative humidity varied between 40% and 50% during the period in which the experiments were carried out. The experimental setup is described in more detail in Kogelheide *et al.* and a scheme of the dielectric barrier discharge can be found in the ESI.^56^ The electrode was driven with a pulsed power supply.^57^ For the experiments in this study the repetition frequency was set to 300 Hz and the amplitude of the HV pulse to 24 kV_pp_. The dielectric barrier discharge has been characterised regarding several plasma parameters as well as reactive species. In Kogelheide *et al.*, the electron density distribution in the discharge is described in detail.^57^ The radial profiles of the plasma produced oxygen species, atomic oxygen (O) and ozone (O_3_), within the plasma volume of the former used plasma source are determined using two-photon laser-induced fluorescence spectroscopy (TALIF) and optical absorption spectroscopy (OAS) in Baldus *et al.* Furthermore, a model of the afterglow chemistry is described in this paper to obtain insight into the dynamics of the considered reactive oxygen species.^58^

### Materials

All reagents and chemicals were purchased from commercial sources and used without further purification. Ferrocenecarboxylic acid was purchased from ABCR. The chloro(protoporphyrinato)iron(iii) (hemin) complex, Se-(methyl)seleno-l-cysteine, l-selenomethionine, seleno-l-cystine, 1,4,7,10-tetraazacyclododecane-1,4,7,10-tetraacetic acid (DOTA), ZnCl_2_ and *N*,*N*-diisopropylethylamine (DIEA) were purchased from Sigma-Aldrich.

### Preparation of Zn(ii) DOTA complex

To the methanolic suspension (5 ml) of DOTA (40 mg, 0.0989 mmol), *N*,*N*-diisopropylethylamine (DIEA) (51.7 μl, 0.297 mmol) was added directly to the reaction mixture in order to deprotonate carboxylic acid groups. After 30 min, ZnCl_2_ (14.48 mg, 0.0989 mmol) were added at room temperature and stirred under nitrogen for two hours. The solution was opened to air and the reaction was stirred for 12 h. The solution was concentrated. Addition of diethyl ether precipitated a white solid. The product was filtered and washed with cold diethyl ether. After drying a white solid was obtained (0.032 g, 69%). MS (ESI-MS, pos. mode, *m*/*z*): obsv.: 467.88 [M + H]^+^; calcd: 467.78 [M + H]^+^; obsv.: 490.92 [M + Na]^+^; calcd: 490.78 [M + Na]^+^; (ESI-MS, neg. mode, *m*/*z*): obsv.: 466.80 [M − H]^−^; calcd: 466.77 [M − H]^−^; obsv.: 489.88 [M + Na − H]^−^; calcd: 489.78 [M + Na − H]^−^.

### Selenium-containing amino acids sample preparation

The compounds 1, 2 and 3 were dissolved in distilled water with a concentration of 4 mg ml^−1^. Ferrocenecarboxylic acid, chloro(protoporphyrinato)iron(iii) (hemin) and zinc(ii) DOTA complex were dissolved in distilled water with a concentration of 4 mg ml^−1^ with 2 eq. of DIEA. 10 μl were placed on cleaned silicon wafers (Siltronic AG) and treated with the DBD for 1, 3, 5 and 20 min. After treatment, samples were filled into reagent tubes and evaporated liquid replenished with distilled water to the concentration of 1 mg ml^−1^ for the analysis *via* mass spectrometry and HPLC. The samples for FTIR spectroscopy were dried by desiccation after the plasma treatment. As controls, another sample was prepared equally, omitting the plasma treatment. Control samples were placed in ambient conditions like the sample treated for the longest time.

### Mass spectrometry

Analysis of samples by ESI-MS follows our standard operating procedure that has been described before.^46,49^ Electron spray ionization (ESI) mass spectra were obtained on an Esquire 6000 mass spectrometer (Bruker). Full mass spectra of the investigated ferrocenecarboxylic acid, chloro(protoporphyrinato)iron(iii) (hemin), zinc(ii) DOTA, substrates 1, 2 and 3 were acquired in both negative-ion and positive-ion mode with the spectrometer equipped with an ion-trap analyser. Three samples of 10 μl treated for the same time were pooled and diluted tenfold with acetonitrile for 300 μl with a final concentration of 1 mg ml^−1^. Instrumental parameters were tuned for each sample. The capillary voltage was set in a range of −22 to 25 V, the spray voltage was between 3.00 and 4.50 kV, and a capillary temperature of 180 °C was employed. The mass scan range was from *m*/*z* 50 to 2000 amu, for 20 s scan time. Spectra were acquired using a direct infusion setup with a flow rate of 5 μl min^−1^ with a cone voltage of 20 kV. To determine occurring in-source fragments, which increase the sample complexity without yielding significant additional information, MS/MS spectra were acquired using the same conditions with a collision energy ramp between 2.00 and 4.00 eV. Spectra were deconvoluted and a background of ten times noise (500 counts in positive and 5 counts in negative mode) was subtracted before peak annotation. All experiments were performed in triplicates.

### FTIR spectroscopy

Analysis of samples by FTIR follows our standard operating procedure that has been described before.^46,49^ A Bruker Vertex FTIR-micro spectrometer was used for the analysis of the samples. FTIR spectra were recorded from 750 cm^−1^ to 4000 cm^−1^ with a spectral resolution of 4 cm^−1^. For the FTIR spectroscopy of the investigated ferrocenecarboxylic acid, chloro(protoporphyrinato)iron(iii) (hemin), zinc(ii) DOTA, substrates 1, 2 and 3 the 12 spectra were recorded at different positions of each sample with each spectrum representing 32 accumulated spectra. Background spectra were obtained for every samples due to the ambient measurement conditions to compensate water and carbon dioxide content in air. All recorded transmission spectra, *T*, were converted into absorption spectra, *A*:1
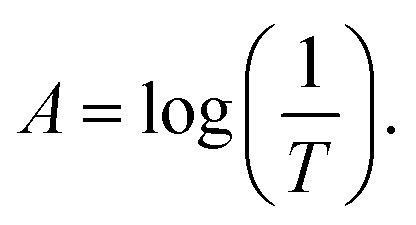


Absorption spectra were baseline corrected afterwards and normalization of the data was carried out applying the Euclidean norm:2
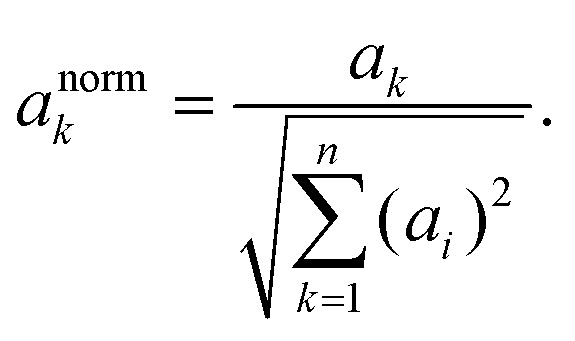


Every data point of each spectrum *a*_*k*_ of wavenumber *k* is normalized to the square root of the sum of every spectrum data point. All experiments were performed in triplicates.

### HPLC

HPLC analysis of samples by HPLC follows our standard operating procedure that has been described before.^49^ An HPLC Knauer system with a quaternary pump and a UV-DAD detector equipped with a Nucleodur® C4 ec column (125 mm × 4 mm, internal diameter 5 μm, Macherey-Nagel), was used. HPLC was performed by using two buffer systems (buffer A: H_2_O/MeCN/TFA, 95 : 5 : 0.1, v/v/v; buffer B: MeCN/H_2_O/TFA, 95 : 5 : 0.1, v/v/v) as the mobile phase. Chromatography was performed with a linear gradient conditions of buffer B (100% in 10 min) from 100% buffer A with a total run time of 50 min. The flow rate of the mobile phase was 1.0 ml min^−1^. 10 μl of the sample was injected. The column was purged with the mobile phase for 2 min, followed by equilibration for 15 min, and then 15 min were required for sample analysis. Spectral data were collected at detection wavelengths of 214 nm and 254 nm, and finally the collected data were plotted.

## Data availability

The authors confirm that all data are available as ESI.† Furthermore, additional data and original files are available from the authors upon reasonable request.

## Author contributions

F. A., D. Ś. and N. M.-N. conceived the experiments. F. A., D. Ś., A. L. S. and F. K. conducted the experiments and analysed the results. F. A., D. Ś., A. L. S., F. K., K. S., P. A. and N. M.-N. wrote and reviewed the manuscript. All authors contributed to the scientific discussion of this work.

## Conflicts of interest

The authors declare that they have no competing interests.

## Supplementary Material

RA-014-D4RA05754F-s001
