# Supplementary material for: Chemical modification of selenium-containing amino acids caused by non-thermal dielectric-barrier discharge atmospheric-pressure plasma
Source: RSC Adv. 2024 Nov 29;14(51):38094–104. doi: 10.1039/d4ra05754f (PMC11606306; doi:10.1039/d4ra05754f)
Supplement: RA-014-D4RA05754F-s001 [file RA-014-D4RA05754F-s001.pdf]

Supplementary Information (SI) for RSC Advances.
This journal is © The Royal Society of Chemistry 2024

Electronic Supporting Information

for

CHEMICAL MODIFICATION OF SELENIUM-CONTAINING AMINO ACIDS CAUSED BY NON-THERMAL DIELECTRIC-BARRIER DISCHARGE ATMOSPHERIC-PRESSURE PLASMA

by

Fahd Afzal[a], Dariusz Śmiłowicz[b], Friederike Kogelheide[c], Anna Lena Schöne[c], Katharina Stapelmann[d], Peter Awakowicz[c], Nils Metzler-Nolte[*,a]

[a] Inorganic Chemistry I – Bioinorganic Chemistry, Faculty of Chemistry and Biochemistry, Ruhr-University Bochum, Bochum, Germany

[b] Department of Chemistry, University of Wisconsin-Madison, Madison, Wisconsin 53706, United States

[c] Institute for Electrical Engineering and Plasma Technology, Ruhr University Bochum, 44780, Bochum, Germany

[d] Department of Nuclear Engineering, North Carolina State University, Raleigh, North Carolina 27695, United States

Contents

1. The scheme of the plasma source p.S2
2. Characterisation of zinc(II) complex p.S2-S4
3. Influence of plasma on zinc(II) complex p.S4-S6
4. Stability experiments of substrates p.S7-S8
5. Influence of plasma on substrates in the presence of iron complexes p.S8-S9
6. Influence of plasma on GSSG in the presence of zinc complex p.S10-S12
7. FT-IR data of substrates after cold plasma treatment p.S13-S15
8. Literature p.S16

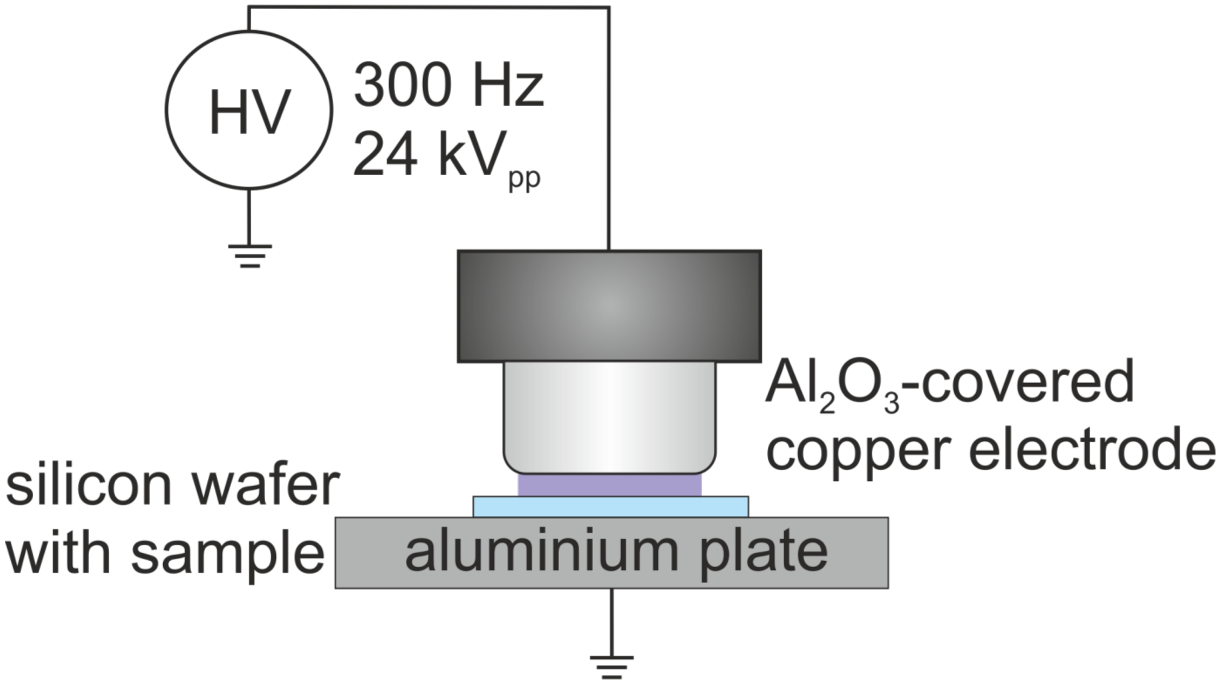

Figure S1. The scheme of the plasma source.[1,2,3]

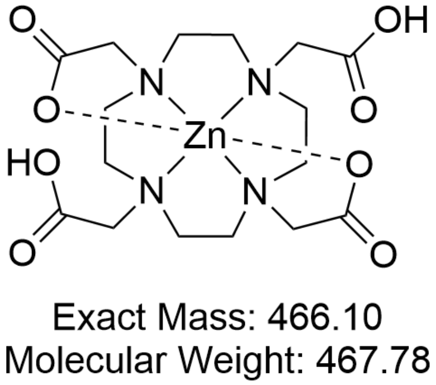

Figure S2. Structure of complex **C**.

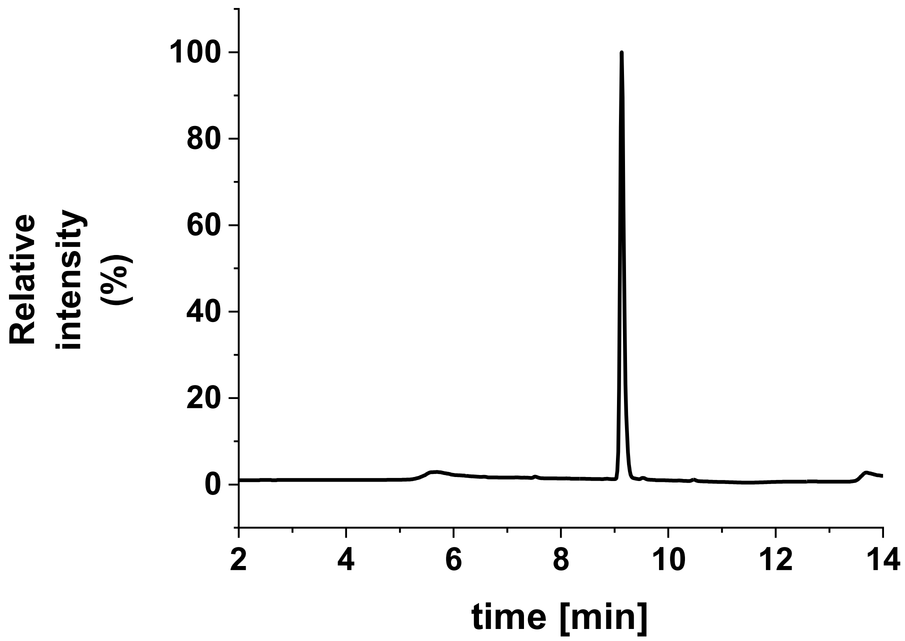

Figure S3. HPLC chromatogram of complex **C**.

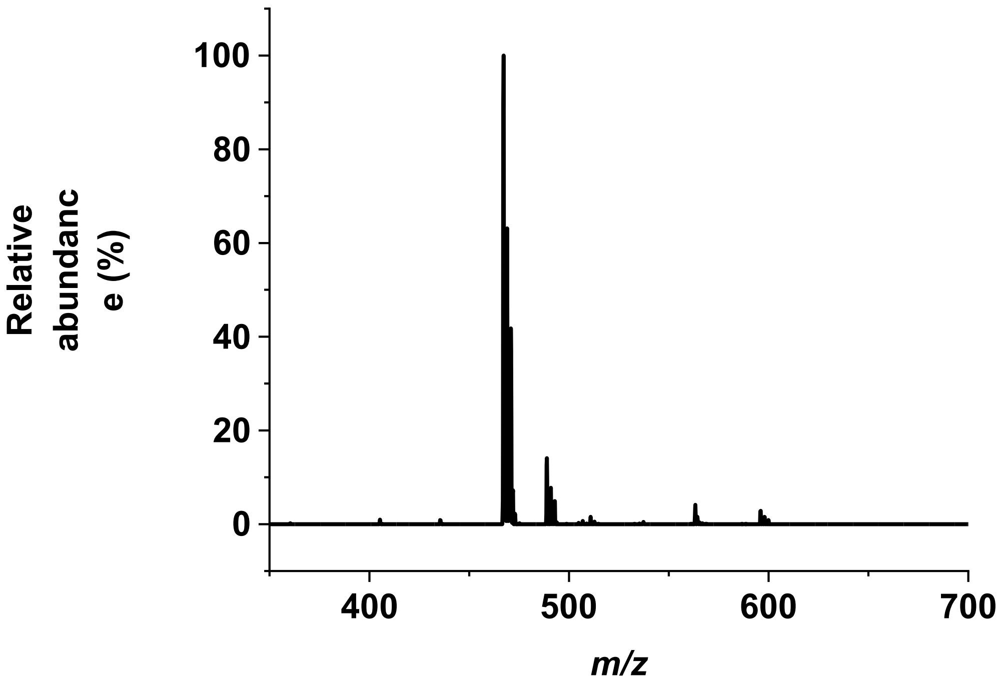

Figure S4. ESI-MS spectrum (positive mode) of complex **C**.

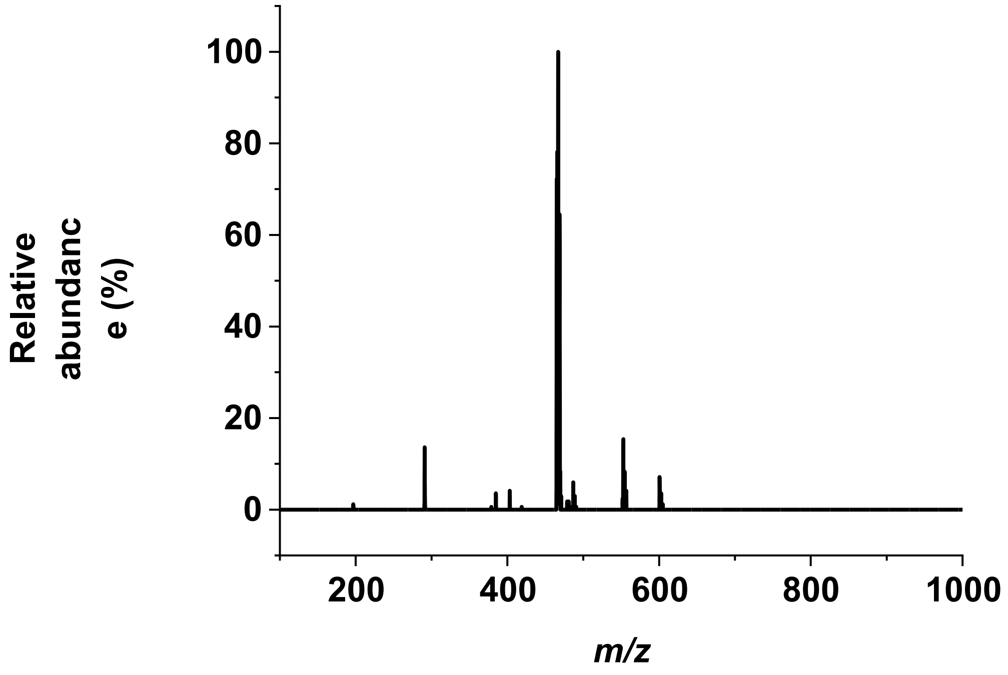

Figure S5. ESI-MS spectrum (negative mode) of complex **C**.

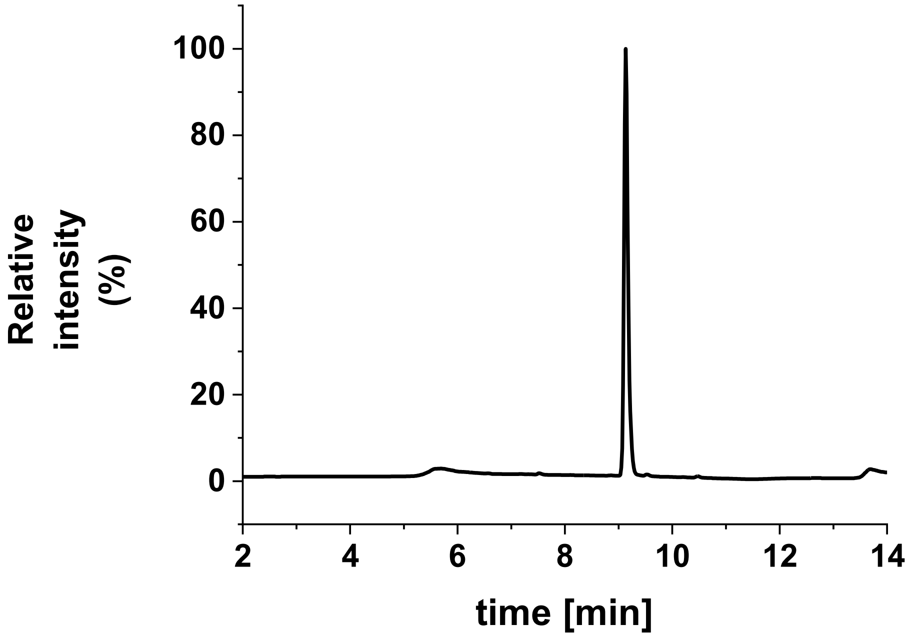

Figure S6. HPLC chromatogram of complex **C** after 2 min of cold plasma treatment.

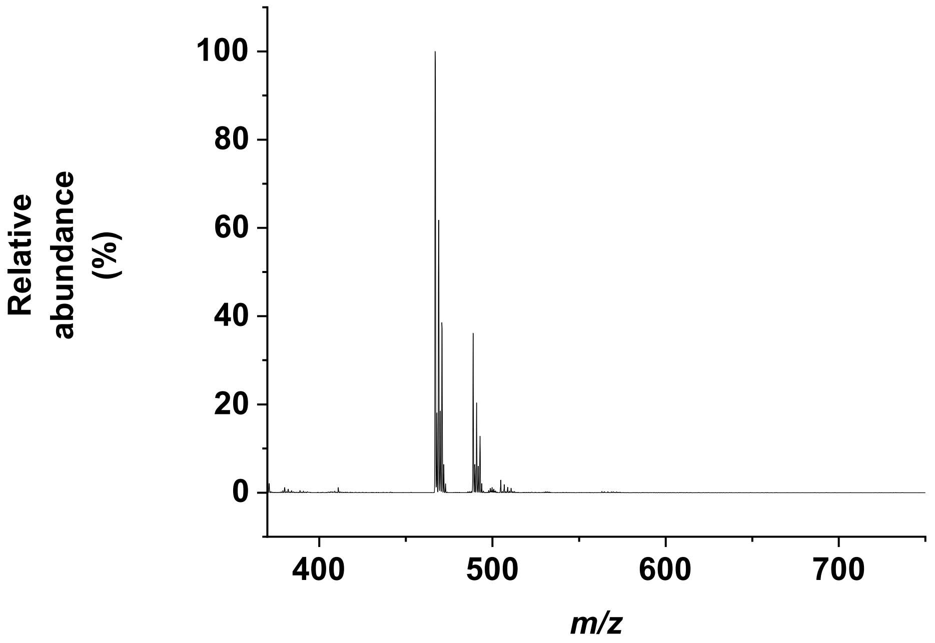

Figure S7. ESI-MS spectrum (positive mode) of complex **C** after 2 min of cold plasma treatment

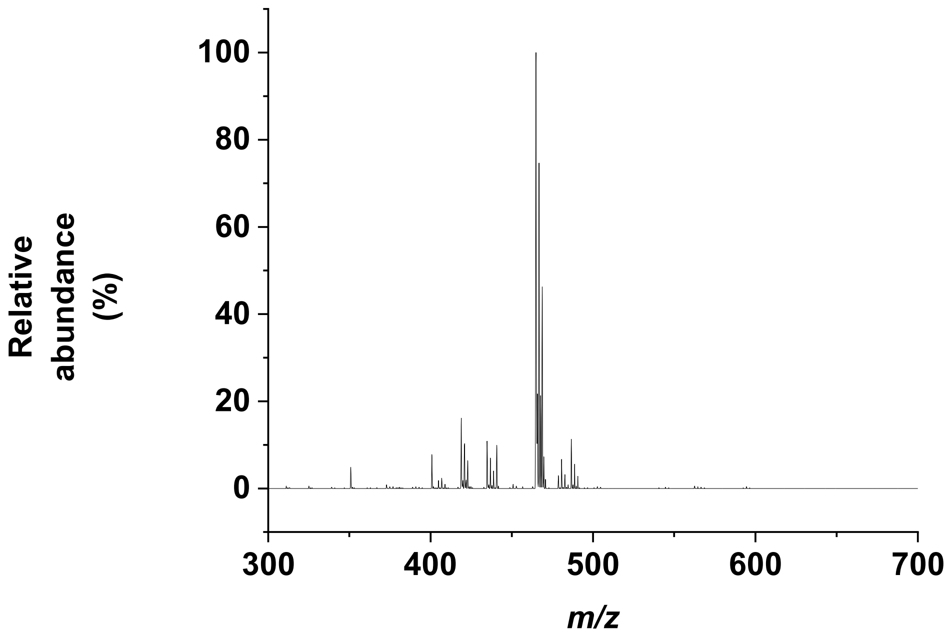

Figure S8. ESI-MS spectrum (negative mode) of complex **C** after 2 min of cold plasma treatment.

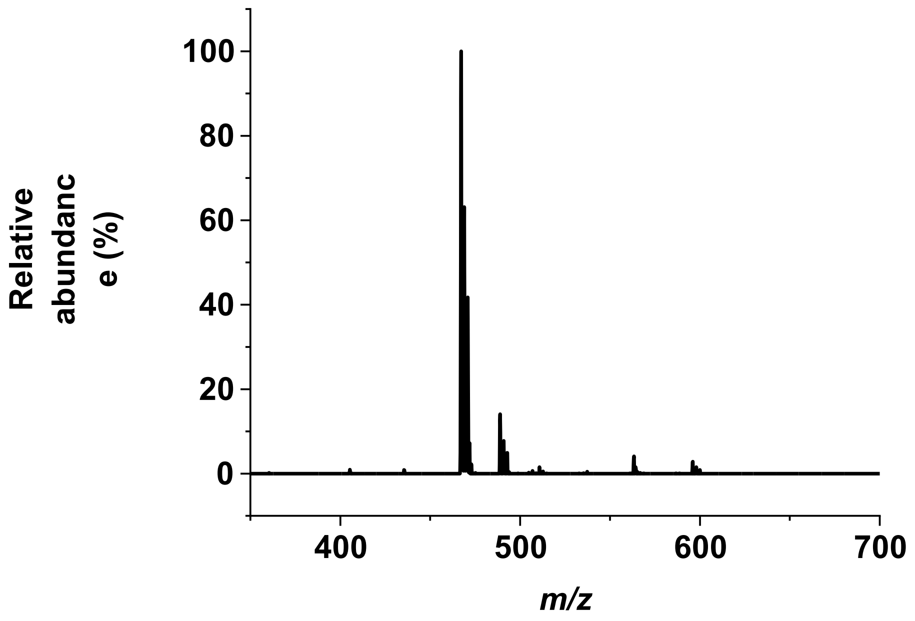

Figure S9. ESI-MS spectrum (positive mode) of complex **C** after 20 min of cold plasma treatment.

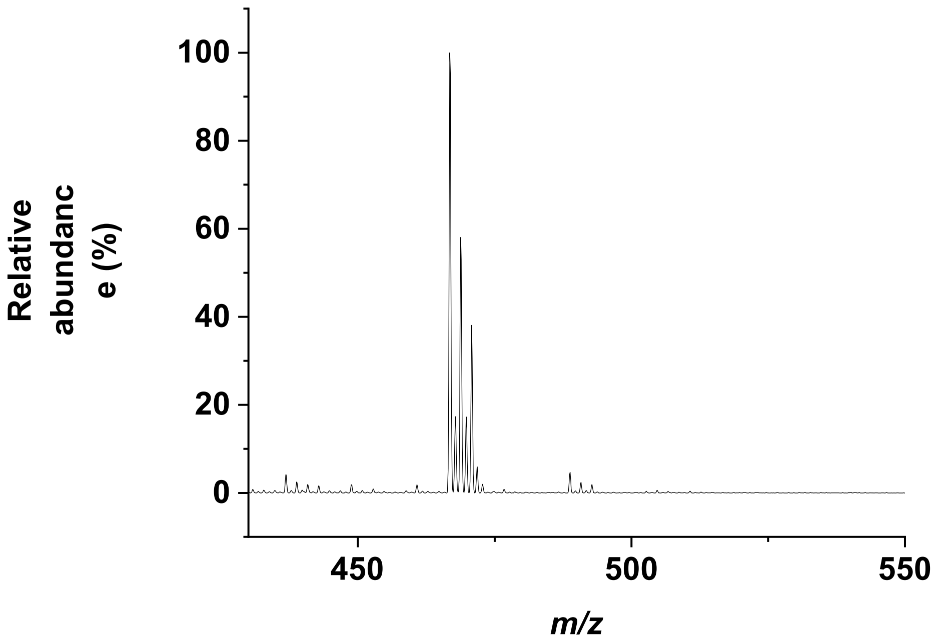

Figure S10. ESI-MS spectrum (negative mode) of complex **C** after 20 min of cold plasma treatment.

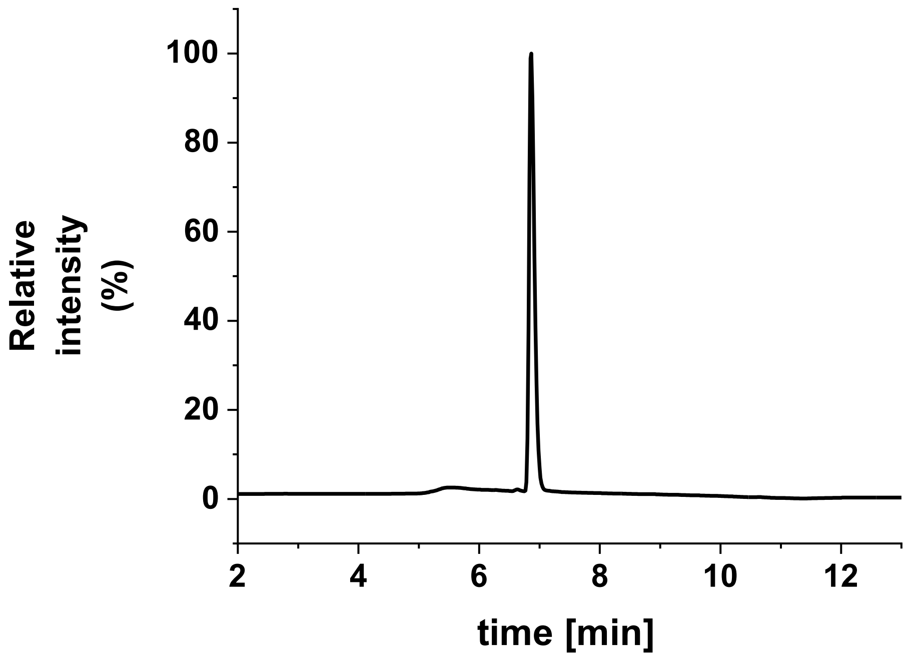

Figure S11. HPLC chromatogram of compound **1** after 5 min of stability experiments.

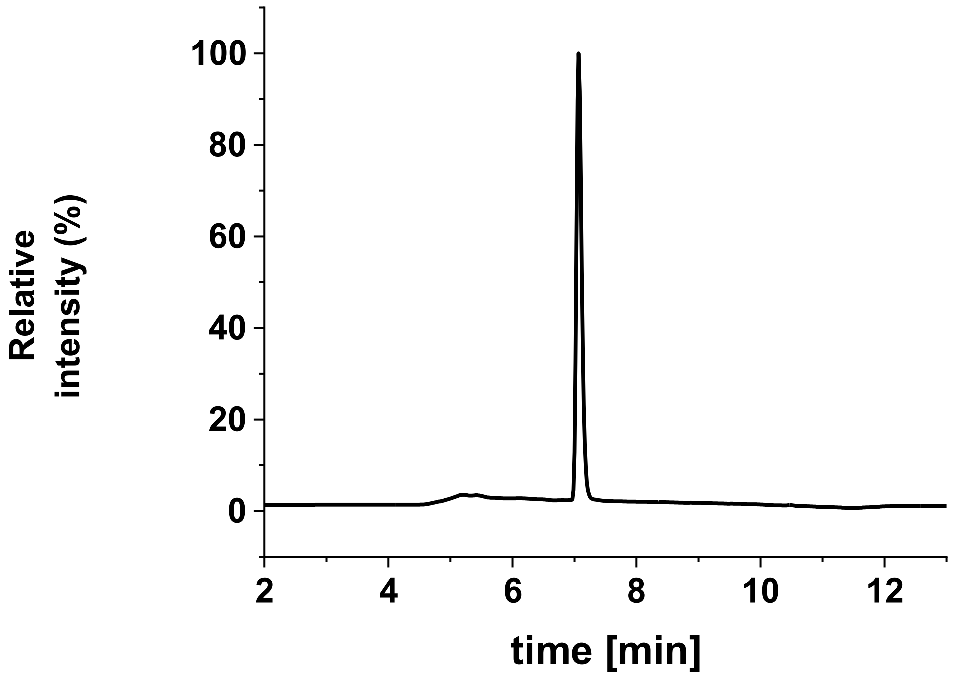

Figure S12. HPLC chromatogram of compound **2** after 5 min of stability experiments.

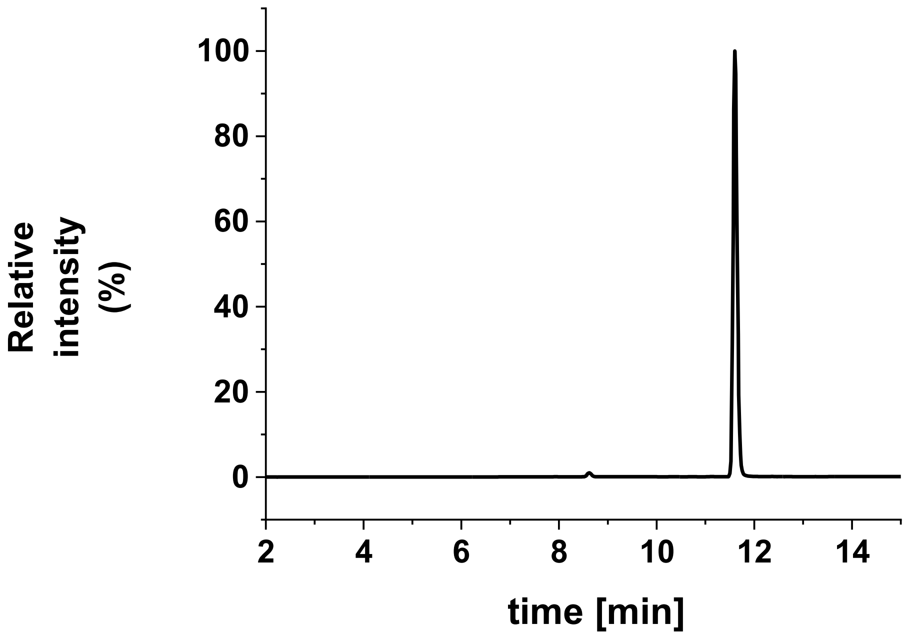

Figure S13. HPLC chromatogram of compound **3** after 5 min of stability experiments.

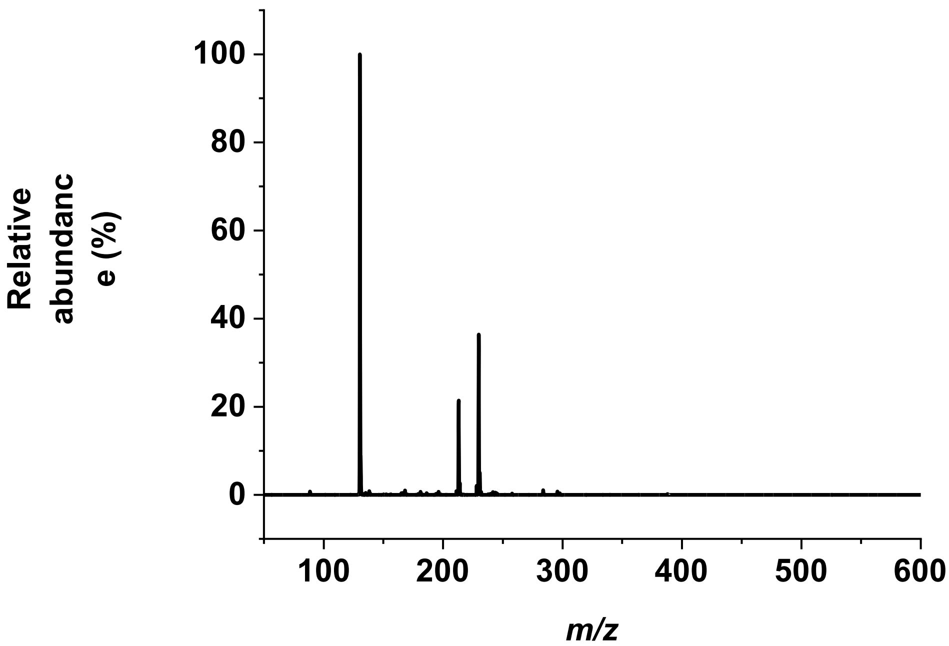

Figure S14. ESI-MS spectrum (negative mode) of compound **1** in the presence of iron(III) after 1 min of cold plasma treatment.

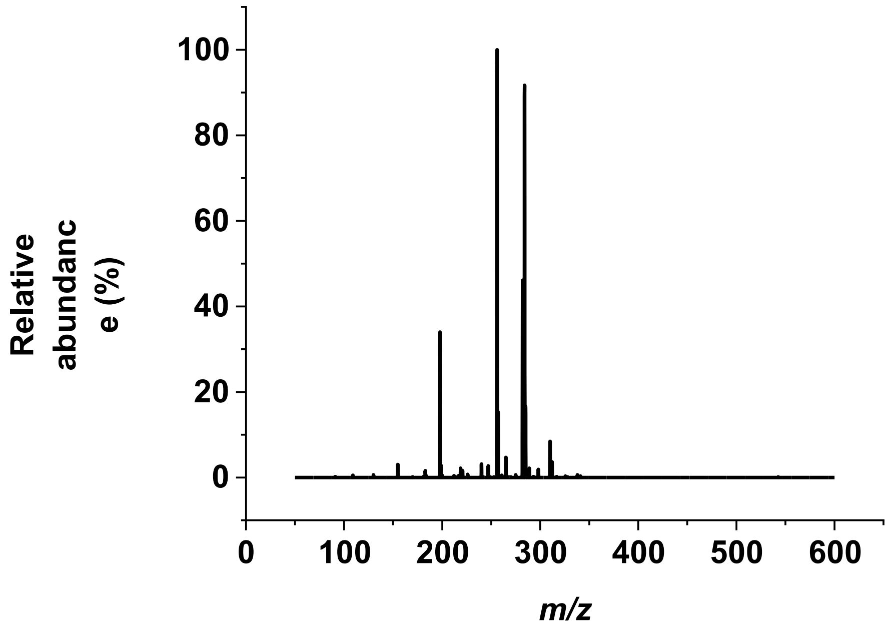

Figure S15. ESI-MS spectrum (negative mode) of compound **3** alone after 3 min of cold plasma treatment.

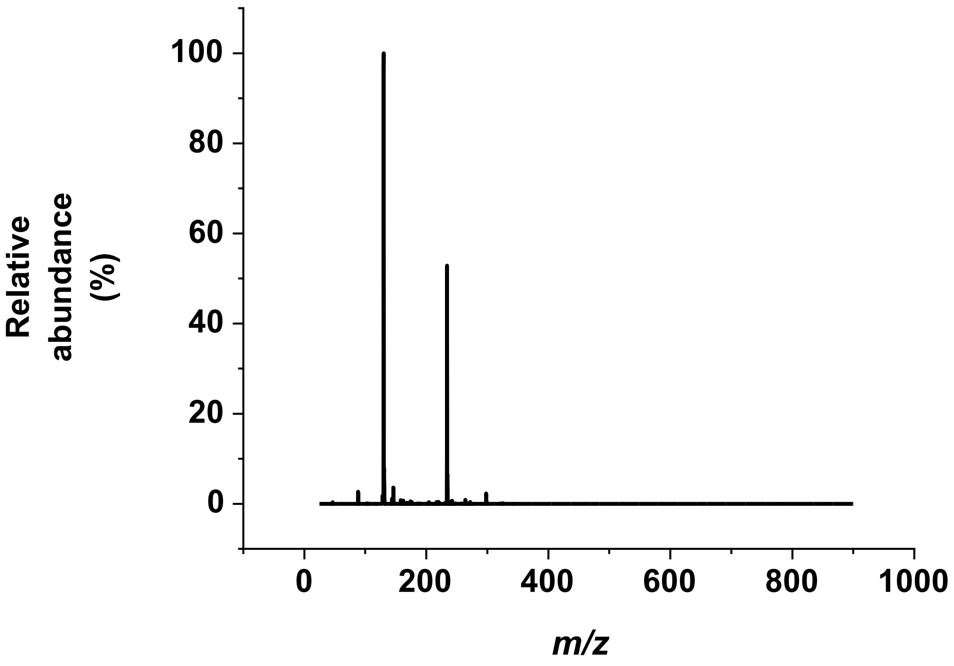

Figure S16. ESI-MS spectrum (negative mode) of compound **3** in the presence of iron(III) after 3 min of cold plasma treatment.

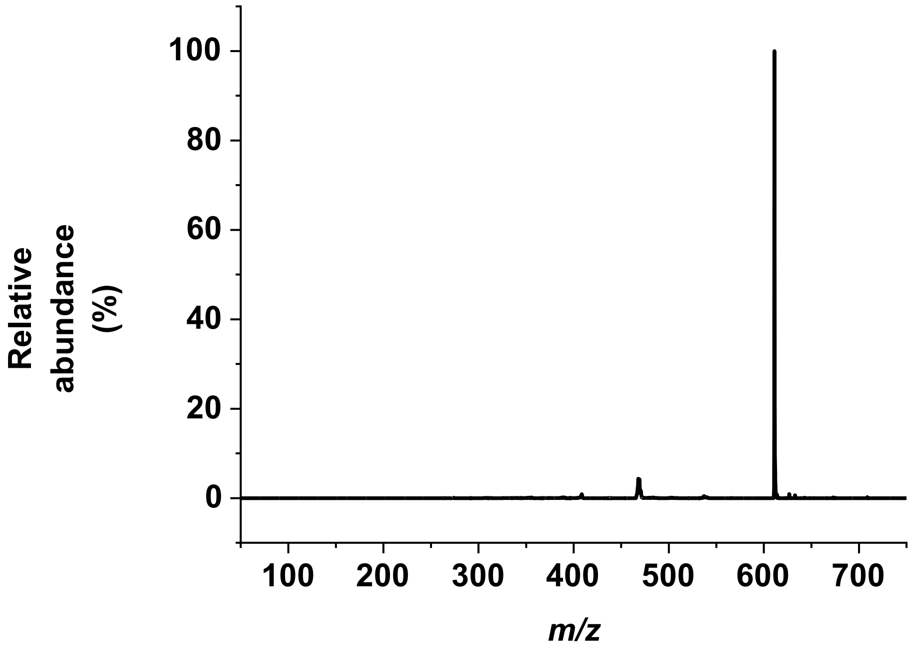

Figure S17. ESI-MS spectrum (negative mode) of GSSG.

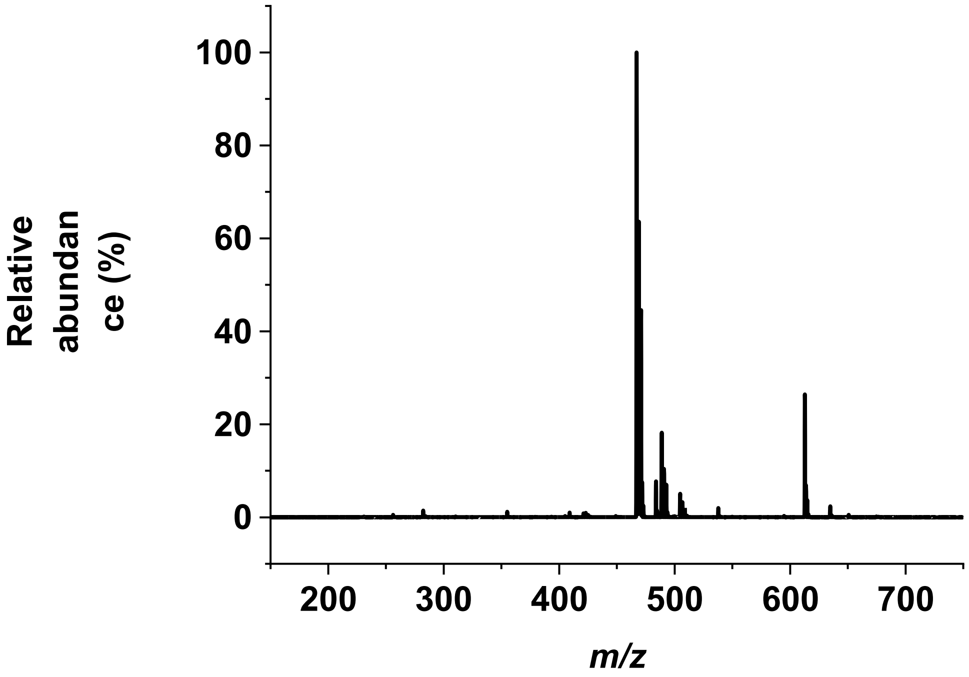

Figure S18. ESI-MS spectrum (positive mode) of GSSG in the presence of zinc(II) complex before cold plasma treatment.

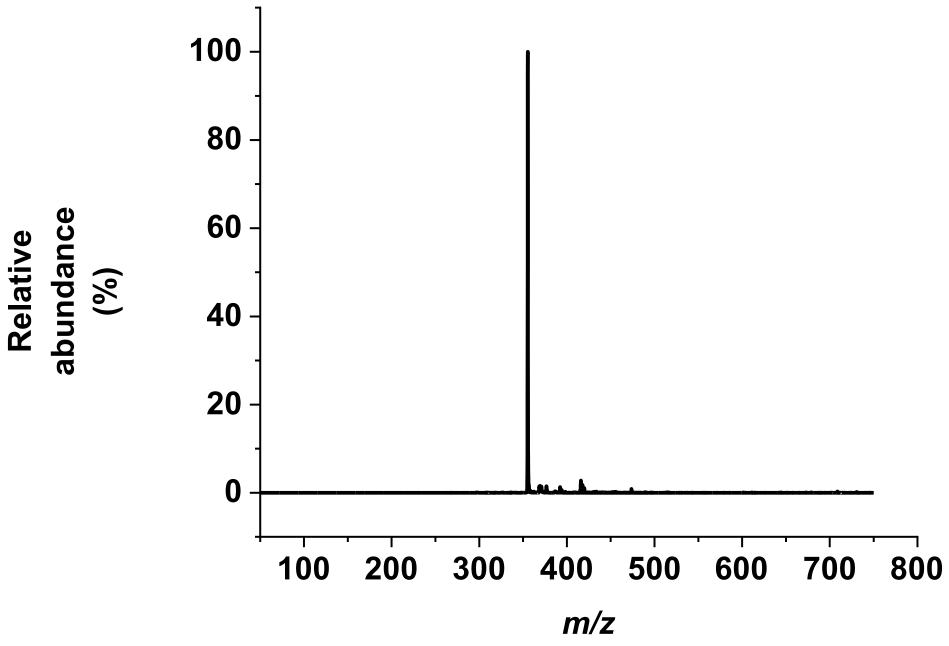

Figure S19. ESI-MS spectrum (negative mode) of GSSG in the presence of zinc(II) complex after 1 min of cold plasma treatment.

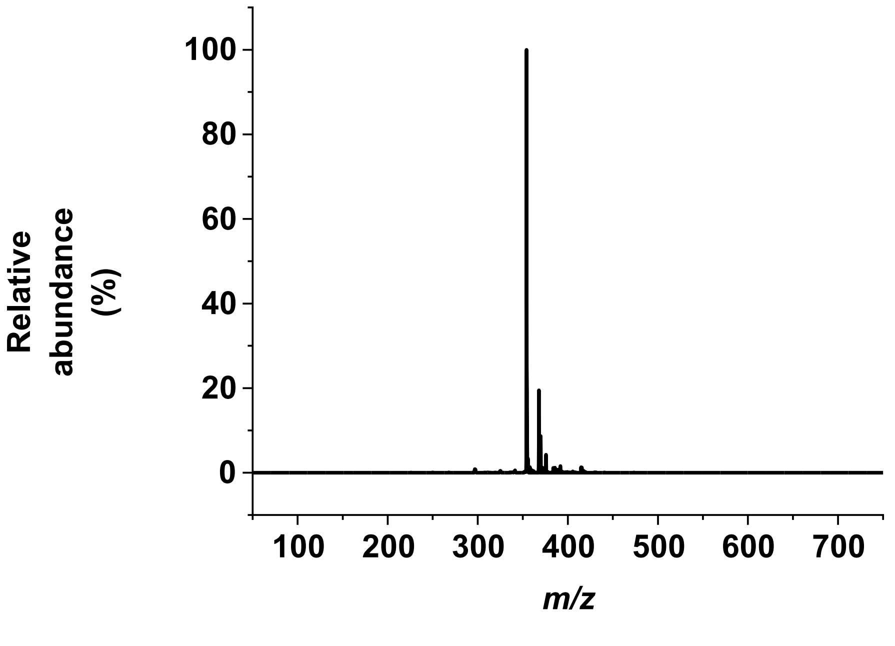

Figure S20. ESI-MS spectrum (negative mode) of GSSG in the presence of zinc(II) complex after 3 min of cold plasma treatment.

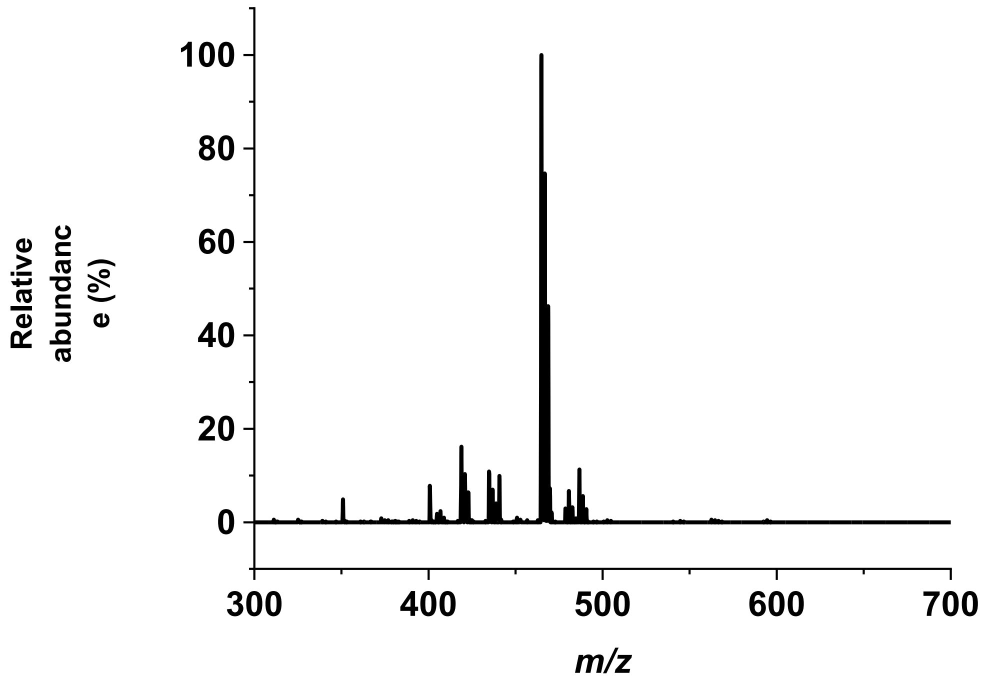

Figure S21. ESI-MS spectrum (negative mode) of GSSG in the presence of zinc(II) complex after 5 min of cold plasma treatment.

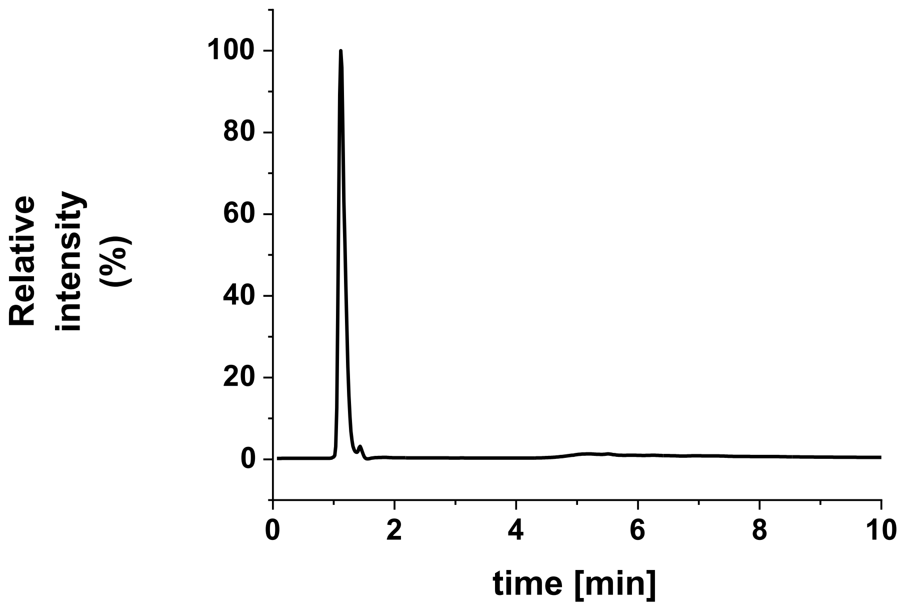

Figure S22. HPLC chromatogram of GSSG in the presence of zinc(II) complex after 5 min of cold plasma treatment.

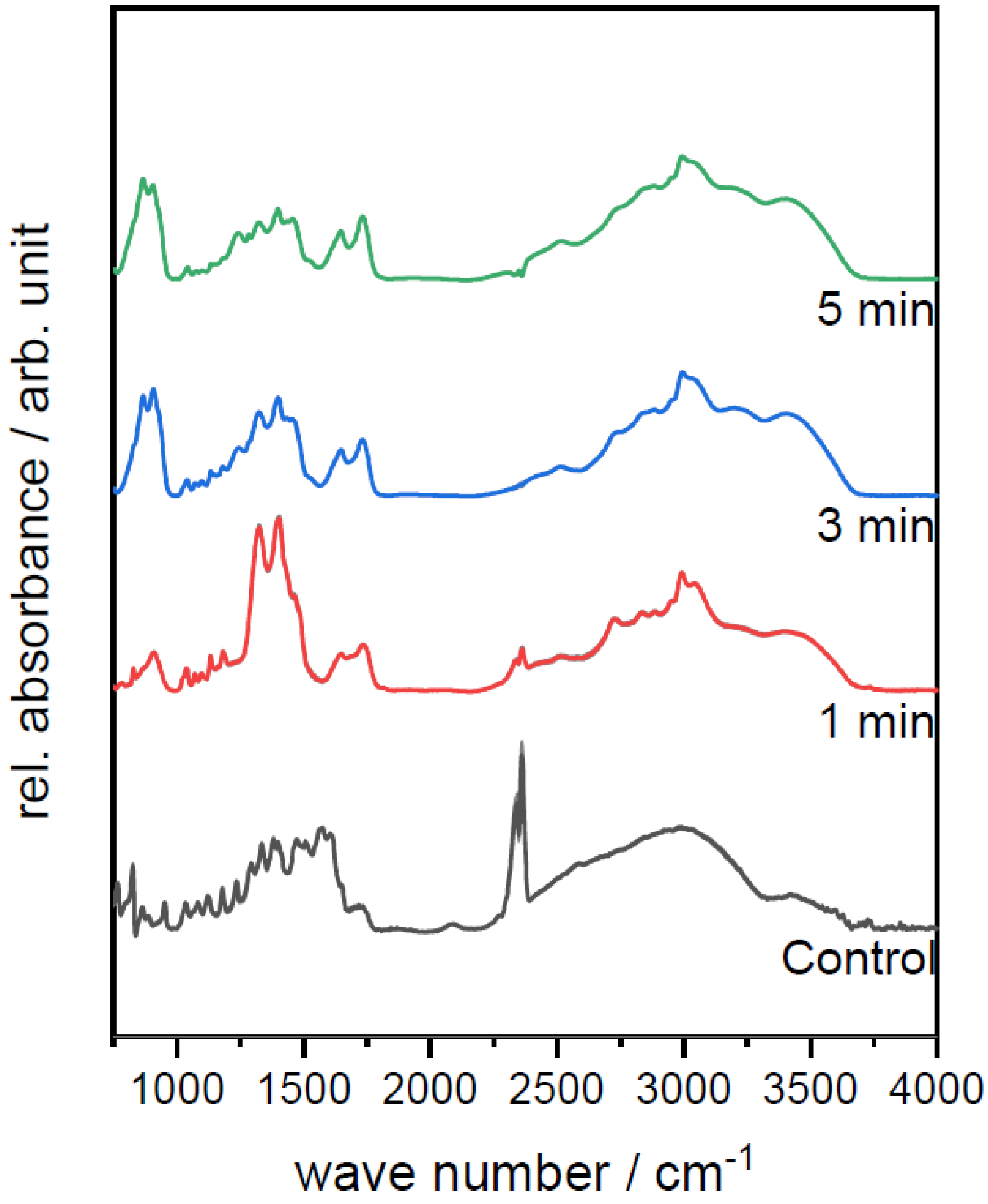

Figure S23. Mean FTIR-spectra of plasma-treated compound **3** in the range of 700-4000 cm^{-1} as a function of different treatment times. Standard deviation of the mean is shown as grey area at each graph**.**

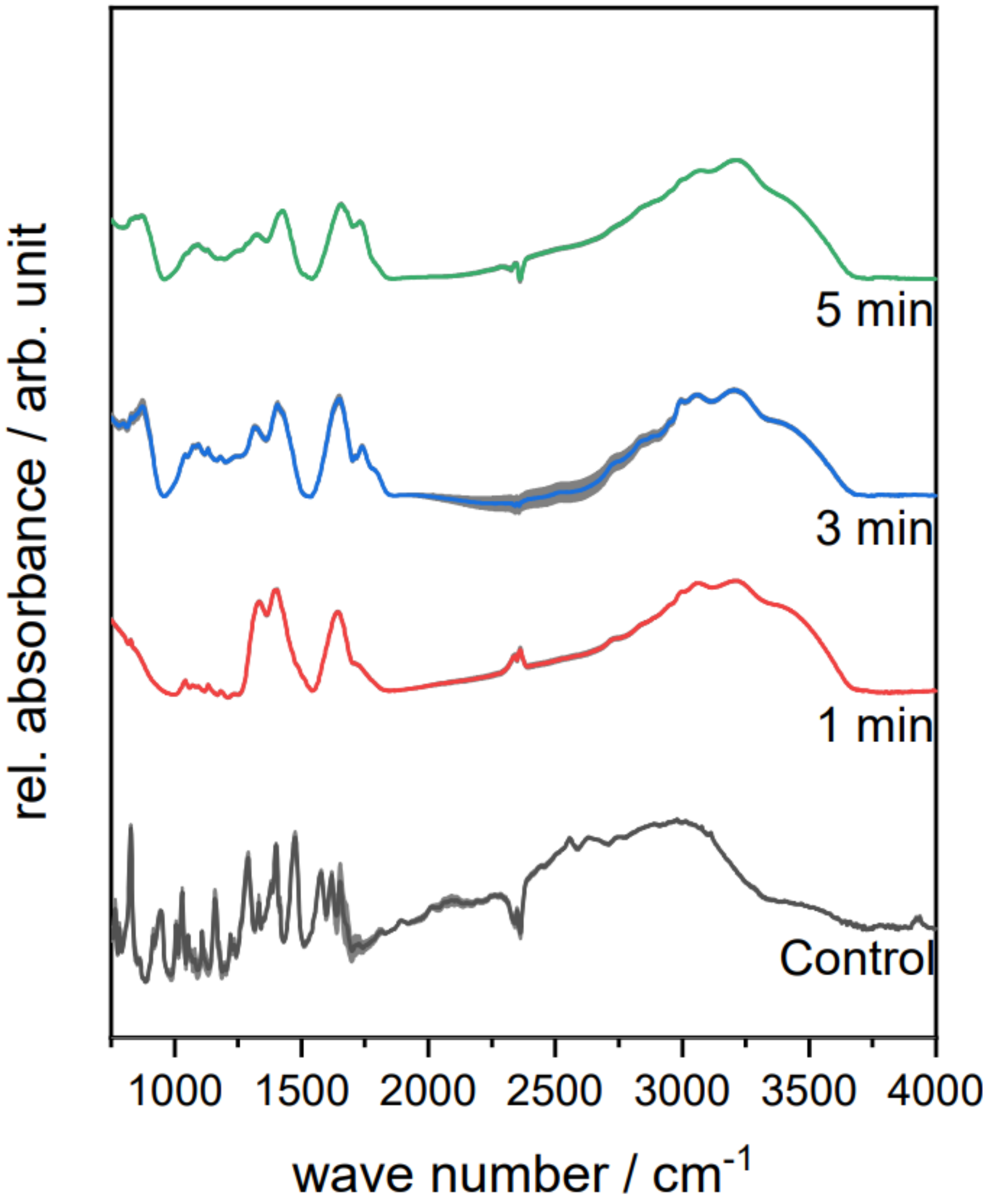

Figure S24. Mean FTIR-spectra of plasma-treated compound **3** in the presence of complex **A** in the range of 700-4000 cm^{-1} as a function of different treatment times.

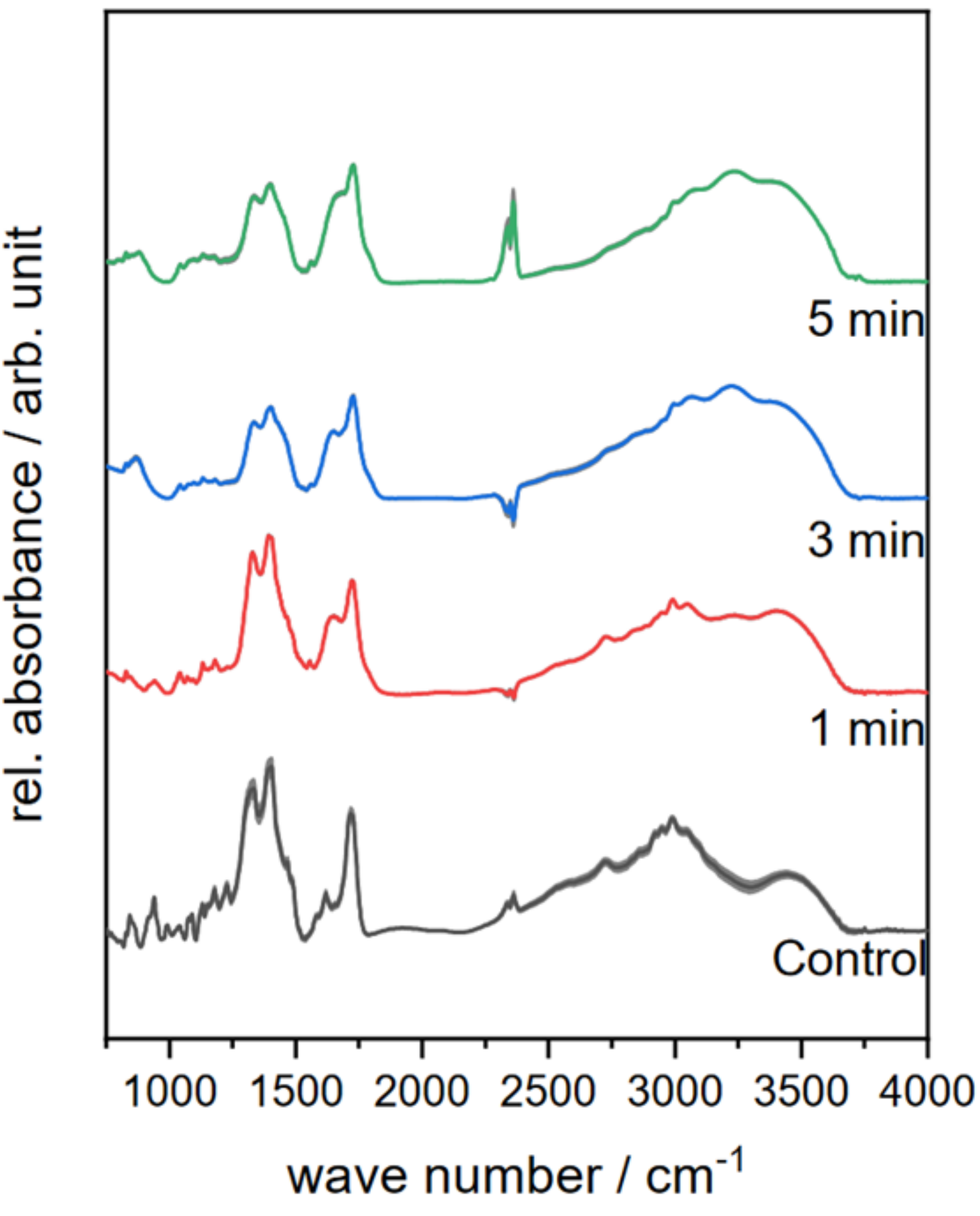

Figure S25. Mean FTIR-spectra of plasma-treated compound **3** in the presence of complex **B** in the range of 700-4000 cm^{-1} as a function of different treatment times.

References

1. Kogelheide, F.; Kartaschew, K.; Strack, M.; Baldus, S.; Metzler-Nolte, N.; Havenith, M.; Awakowicz, P.; Stapelmann, K.; Lackmann, J.-W., FTIR spectroscopy of cysteine as a ready-to-use method for the investigation of plasma-induced chemical modifications of macromolecules. *Journal of Physics D: Applied Physics* **2016,** *49* (8), 084004.
2. Kogelheide, F.; Offerhaus, B.; Bibinov, N.; Krajinski, P.; Schücke, L.; Schulze, J.; Stapelmann, K.; Awakowicz, P., Characterisation of volume and surface dielectric barrier discharges in N2–O2 mixtures using optical emission spectroscopy. *Plasma Processes and Polymers* **2019**, e1900126.
3. Baldus, S.; Schroeder, D.; Bibinov, N.; Schulz-von der Gathen, V.; Awakowicz, P., Atomic oxygen dynamics in an air dielectric barrier discharge: a combined diagnostic and modeling approach. *Journal of Physics D: Applied Physics* **2015,** *48* (27), 275203.
